# Retrospective Outcome Monitoring of ADHD and Nutrition (ROMAN): The Effectiveness of the Few-Foods Diet in General Practice

**DOI:** 10.3389/fpsyt.2020.00096

**Published:** 2020-03-12

**Authors:** Lidy Pelsser, Klaas Frankena, Jan Toorman, Rob Rodrigues Pereira

**Affiliations:** ^1^ADHD Research Centre, Eindhoven, Netherlands; ^2^Adaptation Physiology group, Wageningen University & Research, Wageningen, Netherlands; ^3^Retired, Eindhoven, Netherlands; ^4^Medical Center Kinderplein, Rotterdam, Netherlands

**Keywords:** attention-deficit/hyperactivity disorder, nutrition, few-foods, diet, children, oppositional defiant disorder, prevention, food-induced

## Abstract

**Introduction:**

Double-blind placebo-controlled studies investigating the effect of a few-foods diet (FFD) on attention-deficit/hyperactivity disorder (ADHD) have provided consistent evidence that ADHD can be triggered by foods, indicating the existence of a food-induced ADHD subtype. In 2001 the “few-foods” approach was included in an ADHD treatment protocol. This approach consists of (a) determining, by means of an FFD, whether food is a trigger of ADHD; (b) reintroducing, in FFD responders, foods to assess which foods are incriminated; (c) finally composing a personalised diet eliminating the involved foods only. In the Netherlands the few-foods approach is applied in practice. We aimed to retrospectively assess its effectiveness on ADHD and oppositional defiant disorder (ODD) in real life.

**Methods:**

Data from all children who started the few-foods approach in three specialised healthcare facilities during three consecutive months were included. Behavior was assessed at start and end of the 5-week FFD, using the ADHD Rating Scale and a structured psychiatric interview. Clinical responders (behavioral improvements ≥40%) proceeded with the reintroduction phase.

**Results:**

Data of 57 children, 27 taking medication and 15 following some elimination diet at start, were available. No differences were noted between parental scores of children with and without medication or some elimination diet at start. 21/27 (78%) children stopped taking medication during the FFD. 34/57 (60%) children were ADHD responders, 20/29 (65%) children meeting ODD criteria were ODD responders. 26/34 (76%) ADHD responders started the reintroduction phase; 14/26 (54%) still participated at six months. Teacher data were available of 18/57 (32%) children. 9/18 (50%) children were ADHD responders.

**Conclusion:**

The FFD, if applied by trained specialists, may lead to clinically relevant reduction of ADHD and ODD symptoms in general practice, and a concomitant decrease of ADHD medication. These results corroborate the existence of an ADHD subgroup with food-induced ADHD. Defining and eliminating the incriminated foods, i.e. the underlying causal triggers, may result in secondary prevention of food-induced ADHD. Research into underlying mechanism(s) is of vital importance: finding an easier method or biomarkers for diagnosing food-induced ADHD and ascertaining the incriminated foods may lead to redundancy of the few-foods approach.

## Introduction

Attention-deficit/hyperactivity disorder (ADHD), a mental disorder with a worldwide prevalence estimate of 5–7% (1), is characterized by impairing symptoms of inattention, impulsive behavior and hyperactivity (2). ADHD is a complex condition in which both genetic (3) and environmental factors (4) are involved. The precise causes of ADHD are still unknown, resulting in therapy aimed at fighting symptoms rather than the underlying cause (4). Currently, ADHD treatment advice consists of non-pharmacological interventions complemented with medication if necessary (4). However, the effects of behavioral treatments are small to modest (5), while the effect of the most prescribed ADHD medication (methylphenidate) (6) is limited—being optimal during school hours, but not in the early mornings or evenings. Also, the long-term effect of medication has not yet been established (7), indicating that the need for other therapies, preferably aimed at the underlying triggers of ADHD, is urgent.

Diet has been an environmental factor of long-standing interest in ADHD. Diet research has predominantly focussed on food additives, fish oil and on the few-foods diet (FFD), a very restricted elimination diet consisting of a few foods only. Meta-analyses including double-blind placebo-controlled (DBPC) studies only showed that the clinical effects of fish oil and food additives were negligible to modest, while the effects of the FFD on ADHD were substantial (8), pointing to the existence of a food-induced subtype of ADHD. Subsequent single-blinded (9) and open (10, 11) randomised controlled trials (RCT) investigating the effect of an optimal FFD—not having to moderate the diet as a consequence of the blinded design [see supplement S1Text (8)]—resulted in clinically relevant effect sizes as well.

In 2001 the “few-foods” approach—a diagnostic procedure to define whether or not food is a trigger of ADHD by means of an individually composed FFD to be followed during several weeks—was included in an ADHD treatment protocol (12). For diet responders, the few-foods approach continues with a reintroduction phase to define the incriminated foods, i.e., adding one food per week to the few-foods diet. If the behavioral problems do not recur, the food is considered “safe” and can be eaten without restriction. Finally, therapy is based on the results of the reintroduction period, i.e., consisting of an individually composed diet advice (12), eliminating exclusively those foods that triggered ADHD behavior.

The current Retrospective Outcome Monitoring of ADHD and Nutrition (ROMAN) study is a post-RCT study to evaluate and compare the effectiveness of the FFD in three general practices applying the few-foods approach. We aim to investigate whether the results of RCT’s applying an FFD in selected groups of children with ADHD can be obtained in a heterogeneous sample of children with ADHD reflecting the real-life situation. We also made an inventory of the number of children still participating in the reintroduction period after 6 months. To the best of our knowledge this is the first multi-centre effectiveness study reporting the effect of an FFD on ADHD, meticulously describing the few-foods approach in practice.

## Materials and Methods

In the Netherlands, the few-foods approach is implemented in practice by trained health care professionals working in specialised centres. Parents wishing to explore the impact of food on ADHD may register their child *via* www.redcentrum.nl. Criteria to start the few-foods approach in practice are (1) long-term and substantial behavioral problems hampering the child’s development according to the parents and/or meeting the DSM-IV criteria for ADHD or ODD; (2) child’s age 2–16 years; (3) Dutch speaking parents; (4) at least average family structure and parenting consistency, based on parental report; (5) commitment of both parents and child. To establish family commitment parents are asked to discuss the impact of the 5-week FFD on family life prior to starting the few-foods approach and children ≥8 years old must give verbal informed assent. In real-life many children with ADHD suffer from comorbid psychiatric disorders, are taking ADHD medication or follow a variety of elimination diets, not based on the few-foods approach. These conditions are no reason for not starting the few-foods approach.

### Few-Foods Approach

The Dutch few-foods approach in general practice adheres to a stringent protocol and data are collected in accordance with the Declaration of Helsinki. The procedure starts with assessing whether family and child meet the inclusion criteria, followed by an inventory of family history and situation, the child’s medical background and baseline assessments (T0) of the child’s behavior. Subsequently a two-week baseline period follows during which the child adheres to its usual diet and parents keep a one-week extended diary, closely recording the child’s daily activities, eating habits, medication and behavior. Supplements (e.g., fish oil, vitamins) but not psychoactive drugs, if any, have to be discontinued. At the end of the baseline period the behavioral measurements are repeated (T1) and parents receive elaborate feedback concerning potential pitfalls for adhering to the FFD, based on the information that has been provided through the diary. Then the FFD—in the Netherlands and in some studies synonymously denominated “Restricted Elimination Diet” (RED)—is explained, after which the child starts the 5-week FFD preceded by a transitional week during which the diet is gradually adapted. Parents are urgently requested to support their child optimally by adhering to the FFD as well and to remove all foods that are not allowed during the FFD.

During the first 2 weeks a slightly extended FFD is followed, consistent with the few-foods diet procedure applied in previous RCTs (9, 11): in addition to the most stringent FFD small portions of specific foods like wheat, lamb and butter (daily), and corn, potatoes, pear spread, mango and honey (twice a week) are allowed as well. Parents are given a schematic overview of which foods are permitted in which amount and on which days. If no behavioral improvement is reported within two weeks, the diet will be gradually further restricted to the most stringent FFD consisting of rice, turkey, vegetables (cabbage [white, green, Chinese, red], beet, cauliflower, borecole, swede, sprouts, lettuce), pear, olive oil, ghee, salt, and rice drink with added calcium. If parents of children taking psycho-active drugs report improvement of the child’s behavior after two weeks FFD, the medication will be suspended. If not, the diet is further adapted, and suspension of medication will again be discussed at the end of the third and fourth week of the FFD. At the end of the FFD (week 5) the behavioral measurements are repeated (T2).

### Clinical Responders

The further procedure depends on the child’s response to the diet: (1) Children not taking medication at start, not responding favourably to the medication or in whom medication cannot be suspended during the FFD are designated clinical responders when showing behavioral improvements of at least 40% on either the ACS or the ARS at T2 compared to T1. (2) Children taking medication at start and responding favourably, i.e., not showing ADHD behavior at start, are considered clinical responders when at T2 (a) measurements are without medication, (b) children do not meet the DSM-IV ADHD criteria, and (c) the ARS total score is below the 80th percentile (13).

### Reintroduction Phase

Clinical responders, i.e., children with food-induced ADHD, may start the reintroduction phase to define the incriminated foods. Generally, only children showing behavioral improvements of 40% or more at T2 proceed with the reintroduction phase, although some families do not continue due to the strenuous prospects and the lengthy reintroduction procedure. Conversely, some children showing less improvements sometimes proceed if their parents consider the beneficial effect worthwhile. Foods the child missed the most are added first, according to an introduction schedule based on the DBPC studies’ procedures, i.e., one food at a time during a period of one week and at least full servings on day 4–7 (14, 15). The reintroduction phase lasts until the most important foods have been reintroduced, which may take up to 1.5 years, or until parents indicate that they are able to proceed independently. Children who do not respond to the FFD return to their diet as usual.

### Questionnaires

An inventory of the child’s behavior is made using three questionnaires; the Abbreviated Conners’ Scale (ACS) and the ADHD Rating Scale (ARS) at T0, T1, and T2, and a DSM-IV-based structured psychiatric interview to assess Oppositional Defiant Disorder (ODD) at T1 and T2. The questionnaires, which have been described elsewhere (9), use a four-point scale: for the ACS and ARS from twice a week or less (0), more than twice a week but less than once a day (1), once per day (2) to more than once per day (3); for ODD from less than once a week (0), once or twice per week (1), thrice a week (2) to more than thrice a week (3). During each assessment parents and teachers are asked only to take into account the child’s behavior in the week preceding the assessment.

### Informed Consent

In this retrospective study, anonymised data of all children who started the FFD during three consecutive months (November 1, 2012 to February 1, 2013) in three health care centres were included, provided that parents had given written informed consent for retrospective use of the anonymised data. According to the Dutch central committee on research involving human subjects, retrospective studies do not need approval of a medical ethical committee since the data are already available.

### Statistical Analysis

For statistical analysis, STATA version 13 (KF) and SPSS version 15 (LP) were used. Practices were compared regarding the baseline characteristics using Fisher’s exact test for categorical data and F-test for continuous data. To assess the number of children diagnosed with ADHD previous to the start of the few-foods approach children were divided in two age categories, i.e. younger than 8 years old or 8 years and older, since in The Netherlands ADHD research often starts at age of 7, in the 3^rd^ grade (16). All analyses using continuous parent data were by intention to treat, using last observation carried forward. Teacher data analyses were per protocol. Parameters for the differences in behavior between T1 and T2 were % scale reduction and Cohen’s d (effect size).

Considering that in real-life many children with ADHD are taking medication or following some elimination diet and that both medication and dietary eliminations may affect the child’s behavior, differences between groups of children either or not taking medication and either or not following an elimination diet at start were evaluated in both parents’ and teacher’s outcomes, using a paired t-test. Responder versus non-responder and characteristics at start versus diet response or dropout rate analyses were per-protocol and evaluated using Fisher’s exact test.

The effect of health care practice on parental and teacher behavioral outcome scores at T2 (ACS, ARS, ODD), were based on the likelihood ratio test using a general linear model with score at T1 as covariate and with or without practice as independent variable. Fit of these models was evaluated with the link test command of STATA (a significant p-value for the squared predicted value was indicative for lack of fit).

## Results

**Figure 1:**
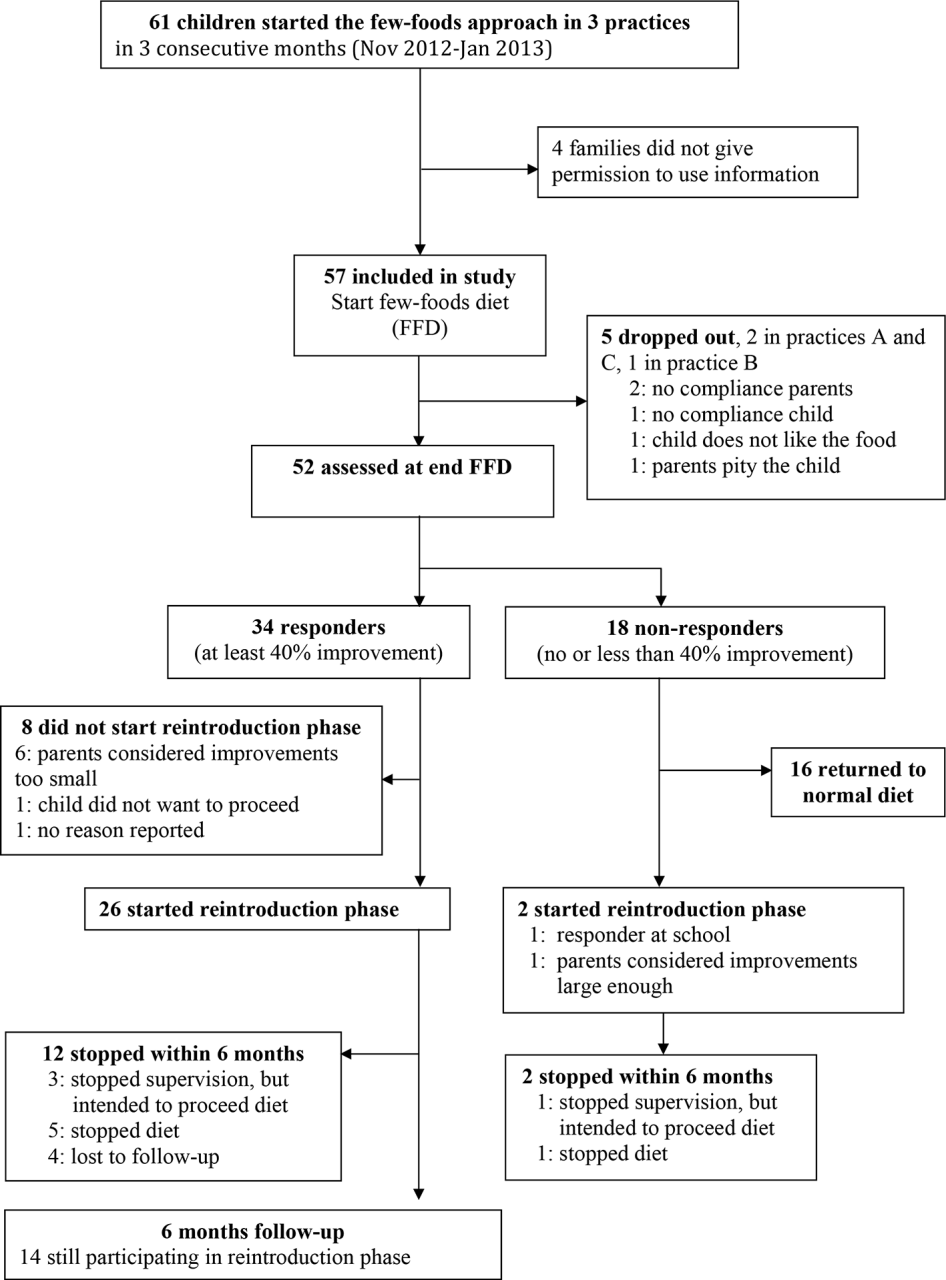
Flow chart study participants.

During three consecutive months 61 children started the few-foods approach in three healthcare centres. Parents of four children did not give informed consent, thus the study cohort included 57 children (**Figure 1**). At start 40/57 (70%) children were already diagnosed ADHD: 2/14 (14%) children younger than 8 years and 38/43 (88%) older children. 29/57 (51%) children met the criteria for ODD. All parents reported serious behavioral problems previous to the start of the few-foods approach. There were no significant differences at baseline between practices (**Table 1**). Teacher measurements were available for 18 of 57 children. Of 39 of 57 children without teacher data, 17 children were not in primary school, 16 teachers were inaccessible (e.g. FFD during holiday, illness teacher) or were unable to cooperate, and 6 parents did not give permission to contact the teacher or stopped the FFD prematurely. 52/57 (91%) children completed the FFD. In 13/52 (25%) children the slightly extended FFD was not adapted; in 18 (35%) the diet was partly adapted, and 21 subjects (40%) followed the most restricted FFD during week 4 and 5 of the FFD.

**Table 1:** Baseline characteristics of the included subjects per practice.

	Practice A N=23	Practice B N=18	Practice C N=16	p value
Boys	18/23 (78%)	12/18 (67%)	14/16 (88%)	0.41
Age	10.6 (3.1)	9.2 (2.9)	8.6 (3.4)	0.14
IQ subject ^#^				
<100	8/18 (44%)	4/13 (31%)	5/10 (50%)	0.54
100-130	10/18 (56%)	8/13 (62%)	4/10 (40%)	
>130	0/18 (0%)	1/13 (8%)	1/10 (10%)	
**Pregnancy and birth**				
Alcohol or smoking during pregnancy	2/23 (9%)	2/18 (11%)	0/16 (0%)	0.55
Problems at birth (hypoxia, incubated)	2/23 (9%)	4/18 (22%)	1/16 (6%)	0.39
**Family data**				
Single parent or co-parenting	3/23 (13%)	1/18 (6%)	2/16 (13%)	0.75
Education parents				
Low^1^	3/23 (13%)	1/18 (6%)	3/16 (19%)	0.79
Middle^2^	12/23 (52%)	11/18 (61%)	7/16 (44%)	
High^3^	8/23 (35%)	6/18 (31%)	6/16 (38%)	
Family structure according to parents				
Good	13/23 (57%)		13/16 (81%)	0.22
Average	10/23 (43%)	4/18 (22%)	3/16 (19%)	
**Subject data ADHD**				
Diagnosed ADHD before start few-foods approach	20/23 (87%)	11/18 (61%)	9/16 (56%)	0.07
Receiving Care as Usual^!^	16/23 (70%)	13/18 (72%)	11/16 (69%)	1.00
Receiving complementary therapy^$^	7/23 (30%)	7/18 (39%)	4/16 (25%)	0.72
Subject following elimination diet*				
Followed diet previous to start few-foods approach	7/23 (30%)	4/18 (22%)	7/16 (44%)	0.46
Still followed elimination diet at start	7/23 (30%)	3/18 (17%)	5/16 (31%)	0.60
**Subject meeting DSM-IV-criteria at start few-foods approach**				
ADHD combined type	14/23 (61%)	10/18 (56%)	4/16 (25%)	0.40
ADHD inattentive type	5/23 (22%)	5/18 (28%)	6/16 (38%)	
ADHD hyperactive-impulsive type	2/23 (9%)	2/18 (11%	4/16 (25%)	
Not meeting ADHD criteria	2/23 (9%)	1/18 (6%)	2/16 (13%)	
Oppositional defiant disorder	12/23 (52%)	9/18 (50%)	8/16 (50%)	1.00
**Subject data psychoactive drugs**				
Taking drugs at start few-foods approach	12/23 (52%)	8/18 (44%)	7/16 (44%)	0.84
Short-acting methylphenidate^@^	4/12 (34%)	3/8 (38%)	3/7 (43%)	1.00
Long-acting methylphenidate	7/12 (58%)	5/8 (62%)	4/7 (57%)	
Dexamphetamine	1/12 (8%)	0/8 (0%)	0/7 (0%)	

Data are number of subjects (%) or mean (SD). ADHD, attention-deficit/hyperactivity disorder. ^#^Data missing of 16 children; IQ never assessed. ^1^Low education=both parents lower vocational education, ^2^middle education=at least one of the parents intermediate vocational education, ^3^high education=at least one of the parents has a university degree.^!^Care as usual=psychoactive drugs and/or psychological interventions (behavioral or cognitive training, parent training). ^$^Complementary therapy=homeopathy, bioresonance, fish oil, supplements. *Child is adhering to some elimination diet prescribed by a therapist or on parents’ own account. ^@^Two children were both on diet and taking methylphenidate at start.

### Comparison at Start

At start of the few-foods approach 27/57 (47%) children were taking psychoactive drugs, while 15/57 (26%) children followed some elimination diet at start, varying from eliminating additives and sugar to eliminating gluten, allergenic foods and/or foods high in histamine. No significant differences were noted between parental behavioral scores of children with and without some elimination diet or medication at start. Significant differences were observed between all teacher’s behavioral scores of children with and without medication at start, e.g. the difference in the ARS total score was -20.7 (95% confidence interval [CI] -31.3 – -10.1; P < 0.001) (**Table 2**). Differences between teacher scores of children with (n=4) and without (n=6) some elimination diet at start were not significant.

**Table 2:** Comparison of behavioral scores at start in children with and without elimination diet (parent ratings) or attention-deficit/hyperactivity disorder (ADHD)–medication (parent and teacher ratings).

	Parent ratings at start in children with (n=15) and without elimination diet (n=42)				Parent ratings at start in children with (n=27) and without ADHD-medication (n=30)				Teacher ratings at start in children with (n=8) and without ADHD-medication (n=10)			
	With diet (SD)	Without diet (SD)	Difference with/without (95% CI)	p value^1^	With medic. (SD)	Without medic. (SD)	Difference with/without (95% CI)	p value^1^	With medic. (SD)	Without medic. (SD)	Difference with/without (95% CI)	p value^1^
**Abbreviated Conners’ scale**												
Score (0-30)	18.1 (6.3)	19.4 (5.4)	-1.2 (-4.6-2.2)	0.48	18.1 (6.1)	19.9 (5.1)	-1.8 (-4.7-1.2)	0.24	9.1 (5.2)	19.3 (4.7)	-10.2 (-15.1– -5.2)	<0.001
**ADHD rating scale**												
Total score (0–54)	33.3 (10.3)	36.9 (9.4)	-3.6 (-9.4–2.2)	0.22	36.3 (10.1)	35.7 (9.4)	0.6 (-4.6–5.8)	0.81	16.4 (11.1)	37.1 (10.1)	-20.7 (-31.3 – -10.1)	<0.001
Inattention score (0–27)	17.9 (6.9)	20.5 (5.3)	-2.6 (-6.1–0.9)	0.14	21.1 (4.9)	18.7 (6.9)	2.4 (-0.7–5.5)	0.13	8.5 (6.8)	20.9 (6.5)	-12.4 (-19.0 – -5.8)	<0.01
Hyperactivity and impulsivity score (0–27)	14.9 (7.0)	16.4 (6.5)	-1.4 (-5.4–2.6)	0.48	15.2 (6.4)	16.7 (6.8)	-1.5 (-5.0–2.0)	0.39	7.9 (5.6)	16.2 (7.9)	-8.3 (-15.4 – -1.3)	<0.05

Data are mean (SD). ADHD, attention-deficit/hyperactivity disorder; ^1^t-test. Medic, medication.

### Comparison T2 Versus T1

Comparison of the parent T1 and T2 scores showed a clinically relevant and statistically significant decrease in all scores (**Table 3**), both in children taking or not taking medication at start (**Table 3A**) and following or not following an elimination diet at start (**Table 3B**). The decrease of the ARS total score was 12.4 (95% CI 7.2–17.6; P < 0.0001; ES=1.07) in 27 children taking medication at start – of whom 21 (78%) did not take medication at T2 anymore – and 17.4 (12.1–22.7; P < 0.0001; ES=1.61) in 30 children without medication at start. The differences in ODD scores in children with and without medication at start were 2.0 (95% CI 0.4–3.6; P < 0.05; ES=1.08) and 3.6 (2.7–4.4; P < 0.0001; ES=2.21).

**Table 3:** Parent measurements at start and end of the few-foods diet (last observation carried forward) in groups of children with and without attention-deficit/hyperactivity disorder (ADHD)–medication (Table 3A) and with and without some elimination diet (Table 3B) at start.

	Start	End	Mean difference start-end (95% CI)	p value^1^	%SR	Cohen’s d		Start	End	Mean difference start-end (95% CI)	p value^1^	%SR	Cohen’s d
**Table 3A**	**Children with ADHD-medication at start**							**Children without ADHD-medication at start**					
**Abbreviated Conners’s scale**	(n=27*)						**(n=30)**						
Score (0-30)	18.1 (6.1)	13.3 (7.8)	4.8 (2.2–7.4)	<0.001	26.5	0.69		19.9 (5.1)	9.6 (7.5)	10.3 (7.4–13.1)	<0.0001	51.8	1.61
**ADHD rating scale**	(n=27*)						(n=30*)						
Parent total score (0–54)	36.3 (10.1)	23.9 (12.9)	12.4 (7.2–17.6)	<0.0001	34.2	1.07		35.7 (9.4)	18.3 (12.1)	17.4 (12.1–22.7)	<0.0001	48.7	1.61
Parent inattention score (0–27)	21.1 (4.9)	14.3 (8.0)	6.8 (3.9–9.7)	<0.001	32.2	1.03		18.7 (6.5)	10.1 (6.4)	8.6 (5.9–11.3)	<0.0001	46.0	1.33
Parent hyperactivity and impulsivity score (0–27)	15.2 (6.4)	9.6 (6.0)	5.6 (2.9–8.2)	<0.001	36.8	0.90		16.7 (6.8)	7.9 (7.1)	8.8 (5.8–11.8)	<0.0001	52.7	1.27
**Structured psychiatric interview**	(n=11**)						**(n=18)**						
Parent ODD score (0–8)	5.2 (1.4)	3.2 (2.2)	2.0 (0.4–3.6)	<0.05	38.5	1.08		5.2 (1.0)	1.7 (2.0)	3.6 (2.7–4.4)	<0.0001	67.3	2.21
**Table 3B**	**Children with some elimination diet at start**							**Children without some elimination diet at start**					
**Abbreviated Conners’ scale**	**(n=15)**						**(n=42)**						
Score (0-30)	18.1 (6.3)	9.3 (7.3)	8.8 (4.6–13.0)	<0.001	48.6	1.29		19.4 (5.4)	12.1 (8.0)	7.3 (4.9–9.6)	<0.0001	37.6	1.07
**ADHD rating scale**	**(n=15)**						**(n=42)**						
Parent total score (0–54)	33.3 (10.3)	15.9 (10.8)	17.4 (10.1–24.7)	<0.001	52.3	1.65		36.9 (9.4)	22.7 (12.9)	14.2 (9.8–18.6)	<0.0001	38.5	1.26
Parent inattention score (0–27)	17.9 (6.9)	8.7 (5.8)	9.3 (5.6–13.0)	<0.001	51.4	1.44		20.5 (5.3)	13.4 (7.7)	7.2 (4.8–9.5)	<0.0001	34.6	1.07
Parent hyperactivity/ impulsivity score (0–27)	14.9 (7.0)	6.8 (6.5)	8.1 (3.9–12.4)	<0.01	54.4	1.20		16.4 (6.5)	9.4 (6.5)	7.0 (4.6–9.3)	<0.0001	42.7	1.08
**Structured psychiatric interview**	**(n=8)**						**(n=21)**						
Parent ODD score (0–8)	5.1 (0.8)	1.8 (2.2)	3.4 (1.6–5.2)	<0.01	64.7	1.99		5.2 (1.3)	2.4 (2.2)	2.8 (1.9–3.7)	<0.0001	53.8	1.55

Data are mean (SD). ADHD, attention-deficit/hyperactivity disorder; ODD, oppositional defiant disorder; SR, scale reduction. *21of 27 and **7 of 11 children ((78% and 64% respectively) without medication at end. ^1^paired t-test.

**Figure 2:**
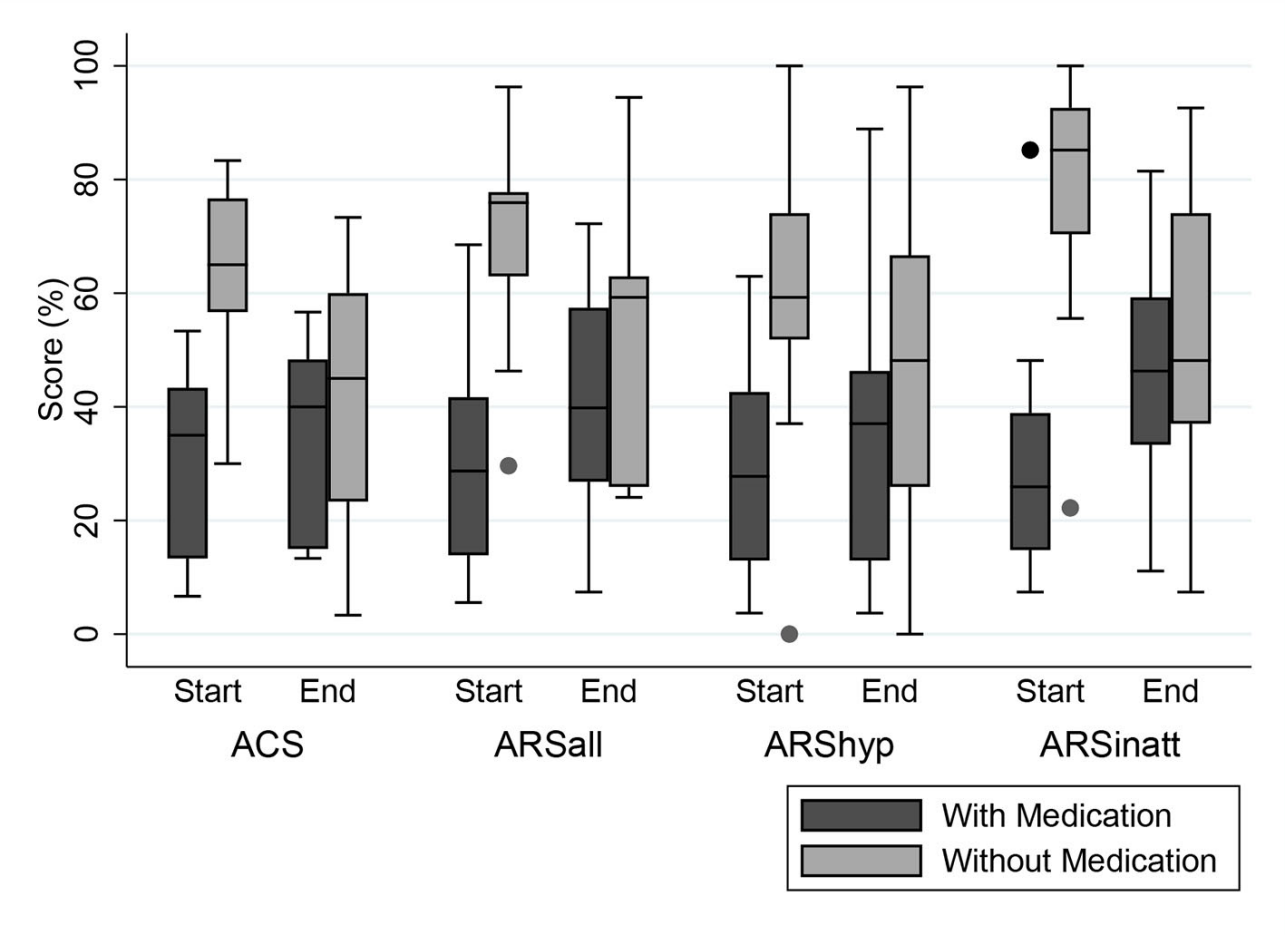
Distribution (Box-Whisker plots) of teacher measurements in percentages of maximum scores at start and end of the 5-week few-foods diet (FFD) in children with (dark grey) and without (light grey) medication at start. Dark grey boxes: 8/18 children taking medication at start (1/8 still taking medication at end). Light grey boxes: 10/18 children not taking medication, neither at start nor at end. To facilitate comparison between the 2 measures, scores have been standardized as percentages of the maximum score per measure. ACS, Abbreviated Conners’ Scale, maximum score 30 (100%). ARS, ADHD Rating Scale. ARSall, total score, maximum score 54 (100%). ARShyp, hyperactivity/impulsivity score, maximum score 27 (100%). ARSinatt, inattention score, maximum score 27 (100%). Shaded boxes denote interquartile range; Horizontal bars within boxes denote median; Whiskers denote 1.5 times the interquartile range, rolled back to an actual data point; Dots are outside values.

Teacher measurements were available for 18 children, eight of whom took medication at start. The ARS total score at the end of the FFD showed a non-significant increase in the children taking medication at start, of whom 7 (88%) stopped medication during the FFD (-5.8 [95%CI -18.9–7.4]; P=0.33; ES=-0.5). In children without medication at start, ARS scores decreased significantly (9.3 [95% CI 2.2–16.4]; P < 0.05; ES=0.8) (**Table 4**). All teacher mean end scores of children taking medication at start were lower than the mean end scores of children without medication at start (**Figure 2**).

**Table 4:** Teacher measurements at start and end of the few-foods diet (per protocol) in groups of children with and without taking medication at start.

	Children with ADHD-medication at start (n=8^*^)						Children without ADHD-medication at start (n=10)					
	Start	End	Mean difference start-end (95% CI)	p value^1^	%SR	Cohen’s d	Start	end	Mean difference start-end (95% CI)	p value^1^	%SR	Cohen’s d
**Abbreviated Conners’ scale**												
Score (0-30)	9.1 (5.2)	10.4 (5.3)	-1.3 (-7.2–4.7)	0.64	-14.3	-0.25	19.3 (4.7)	12.3 (7.5)	7.0 (2.6–11.4)	<0.01	36.3	1.12
**ADHD rating scale**												
Teacher total score (0–54)	16.4 (11.1)	22.1 (11.3)	-5.8 (-18.9–7.4)	0.33	-35.4	-0.51	37.1 (10.1)	27.8 (13.1)	9.3 (2.2–16.4)	<0.05	25.1	0.80
Teacher inattention score (0–27)	8.5 (6.8)	12.5 (5.8)	-4.0 (-9.4–1.4)	0.13	-47.1	-0.63	20.9 (6.5)	14.4 (7.4)	6.5 (2.3–10.7)	<0.01	31.1	0.93
Teacher hyperactivity and impulsivity score (0–27)	7.9 (5.6)	9.6 (7.4)	-1.8 (-10.2–6.7)	0.64	-21.5	-0.26	16.2 (7.9)	13.4 (8.6)	2.8 (-1.1–6.7)	0.14	17.3	0.34

Data are mean (SD). ADHD, attention-deficit/hyperactivity disorder; SR, scale reduction. *7 of 8 (88%) without medication at end. ^1^paired t-test.

### Responders

**Figure 3:**
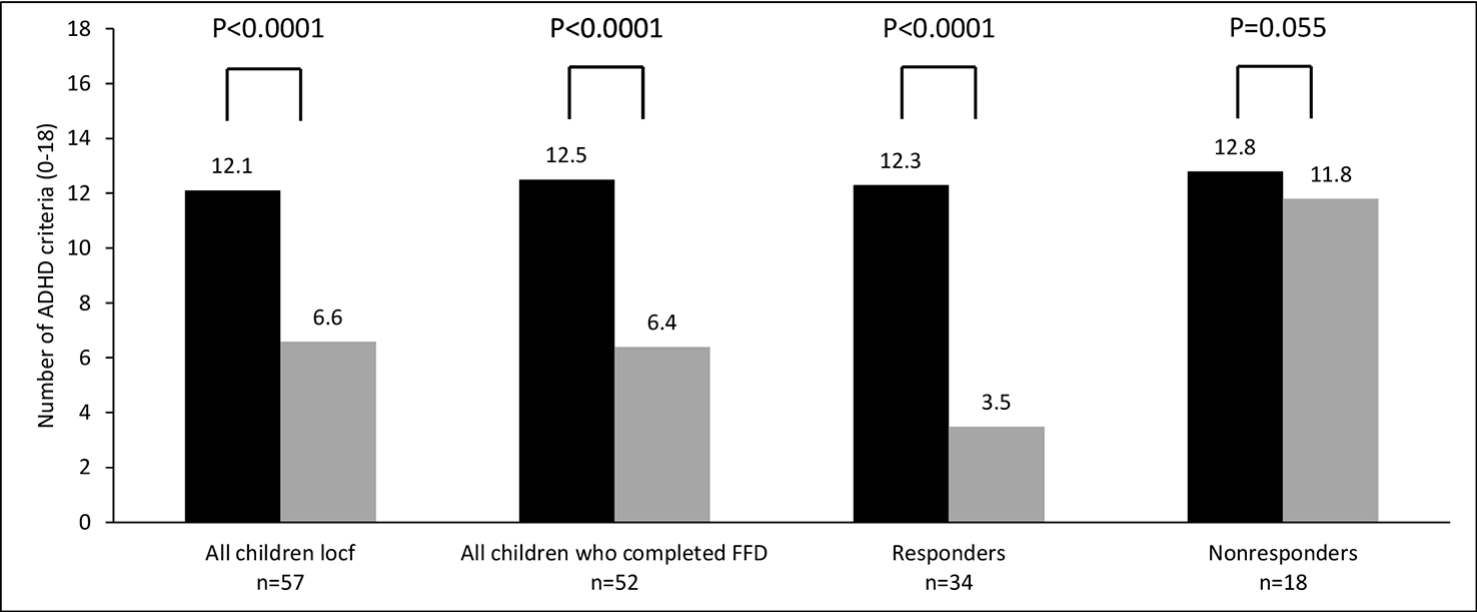
Number of attention-deficit/hyperactivity disorder (ADHD) criteria (0–18) at T1 (black bar) and T2 (grey bar). Locf, last observation carried forward; FFD, few-foods diet; responder, at least 40% improvement; non-responder, no or less than 40% improvement.

**Figure 4:**
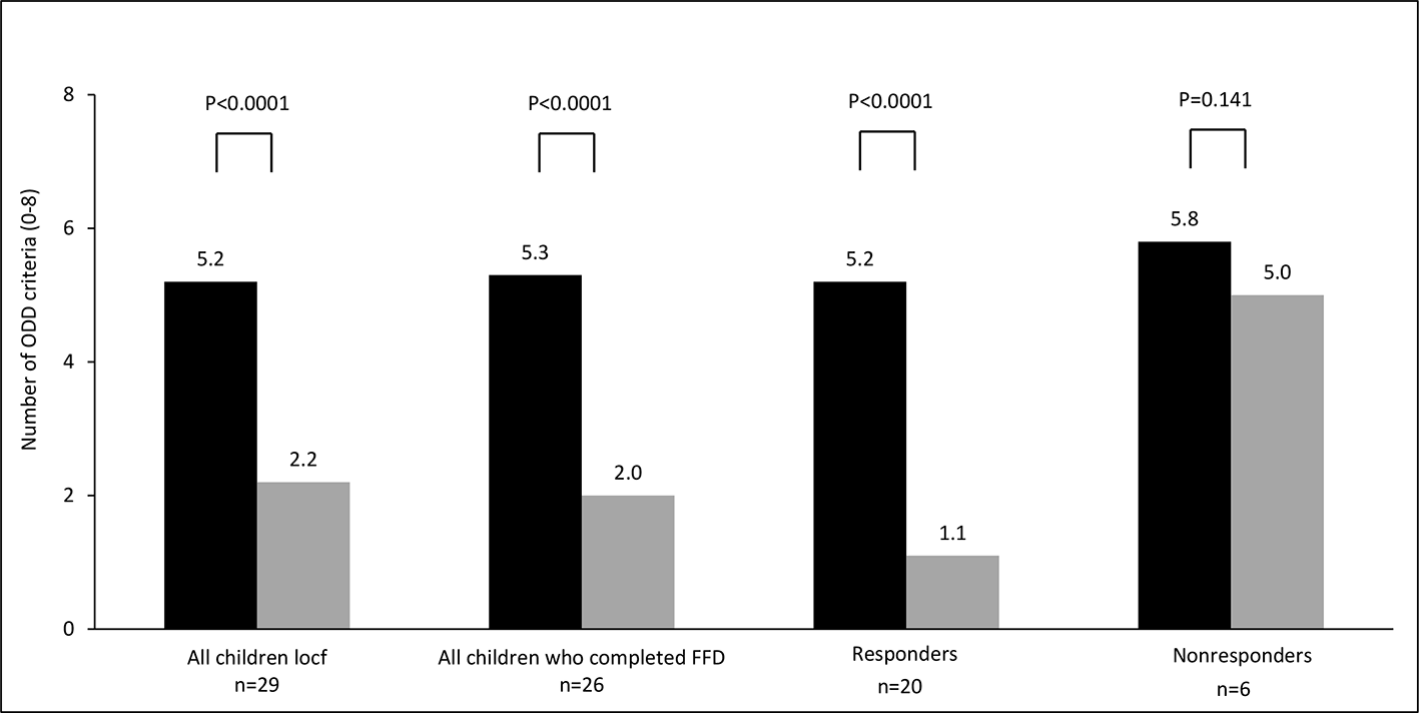
Number of oppositional defiant disorder (ODD) criteria (0–8) at T1 (black bar) and T2 (grey bar). Locf, last observation carried forward; FFD, few-foods diet; responder, at least 40% improvement; non-responder, no or less than 40% improvement.

For all parent measurements the first responder definition (see methods section) was applied, because parental behavioral scores of children taking ADHD-medication or following some elimination diet at T1 were commensurable to the scores of children not taking ADHD-medication or an elimination diet at T1 (**Table 2**). In total 34/57 (60%) children were ADHD responders: 13/27 (48%) children taking medication at start and 21/30 (70%) children without medication at start (P=0.11, Fisher’s exact test) showed behavioral improvements of ≥ 40%. Of the 13 responders taking medication at start 12 (92%) stopped taking medication during the FFD. The end measurements of these children were without medication. 10/15 (67%) children following some elimination diet at start and 24/42 (57%) children without an elimination diet at start (P = 0.56, Fisher’s exact test) were clinical responders. No difference was found in ADHD respondership between age groups (P = 0.25, Fisher’s exact test). The average number of DSM-IV ADHD criteria in responders decreased with 72%, from 12.3 to 3.5 (P < 0.0001) (**Figure 3**). 20/29 (65%) children meeting the ODD-criteria were ODD responders, the average number of criteria decreasing with 79%, from 5.2 to 1.1 (P < 0.0001) (**Figure 4**). 16/20 (80%) ODD responders were ADHD responders as well.

Given the favourable effect of medication on ADHD at school, respondership based on the teacher’s measurements was computed in accordance with the second responder definition. 9/18 (50%) children were clinical responders: 5/8 (63%) children taking medication at start and 4/10 (40%) children without medication at start (P=0.64, Fisher’s exact test). All 5 responders taking medication at start stopped taking medication during the FFD, consequently the end measurements of all responders were without medication.

### Family Structure

In 25/34 (74%) responders good family structure was reported. The remaining 9 families had an average family structure; all nine either did not start the reintroduction phase (n=5) or stopped within 6 months (n=4). 26/34 (76%) responders (22 with good and 4 with average family structure) continued with the reintroduction phase; 14/26 (54%) responders, all reporting a good family structure, still participated at the 6-months’ inventory. Four other families, three with good family structure and one with average structure, stopped supervision within 6 months with the intention to proceed the introductions without supervision. Parental education level had no significant relation with family structure, FFD response or the 6-months’ reintroduction participation. No significant effect (likelihood ratio test) of practice on ACS, ARS and ODD scores at start and end (last observation carried forward) was present. None of the parents or children reported adverse effects during the 5-week FFD or the reintroduction phase. All parents of responders, though relieved by the child’s behavioral improvements and the corresponding amelioration in family life, complained about the long-standing and burdensome reintroduction phase.

## Discussion

The ROMAN study data show that the FFD, applied in general practice by trained physicians, may significantly reduce ADHD and ODD symptoms in children either or not taking medication or following some elimination diet at start. 34/57 (60%) children (parent scores) and 9/18 (50%) children (teacher scores) were ADHD responders. At start 29/57 (51%) children met the criteria for ODD as well: 20/29 (69%) children were ODD responders, 16/20 (80%) children were both ADHD and ODD responder (parent scores).

Children taking medication at start showed significantly less ADHD symptoms at school compared to children not taking medication, while parent scores with and without medication at start were comparable (**Table 2**). This discrepancy is in line with the short-term effect of methylphenidate, the most used ADHD-medication: in early mornings the medication is not yet effective and at evenings its effect is worn out, resulting in recurrence of the ADHD behavior.

In general practice many children with ADHD take medication: in the ROMAN study 27/57 (47%) children took ADHD medication at start of the few-foods approach. However, during the FFD the behavioral improvements were to such an extent that in 12/13 (92%, parent measurements) and 5/5 (100%, teacher measurements) responders, medication was stopped. FFD responders, including the 12/27 (44%) responders who took medication at T1 but not at T2, showed behavioral improvements of 72% (**Figure 3**), indicating that the effect of the FFD on ADHD can be impressive. In light of the limitations of medication, an elimination diet based on the few-foods approach could be the preferential treatment in children with food-induced ADHD (i.e., responding favourably to the FFD), provided that the family is able to complete the reintroduction phase.

No behavioral differences were noted in groups of children with and without some elimination diet at start. However, in both groups the few-foods diet resulted in major decrease of ADHD and ODD symptoms, underlining the importance of applying a short-term and well-monitored few-foods diet instead of “random” elimination of foods. Indeed, in children with ADHD only the effectiveness of the few-foods diet has been well-established: research eliminating additives or sugar did not result in clinically relevant effects (8), while, to the best of our knowledge, the effects of other diets on ADHD—e.g., eliminating allergenic foods, gluten and/or foods high in histamine—have never been investigated. Such haphazardly composed elimination diets may at best have a suboptimal result and should not be recommended, like suboptimal doses of methylphenidate are not recommended either (17).

52/57 (91%) children completed the FFD, which is a considerable number taking the impact of the intervention into account. The low attrition rate might be due to the FFD procedure, allowing some additional foods in the first weeks. Gradually restricting the diet is an important strategic move to help families to persevere when being faced with the immense difference between their regular diet and the FFD. The low attrition rate might also be attributable to the personalised support during the 5-week FFD, based on potential pitfalls recognised in the baseline diary provided by the parents. Although otherwise suggested in ADHD guidelines, see https://www.nice.org.uk/guidance/ng87/chapter/Recommendations#managing-adhd chapter 1.6.3 (18), there is no evidence for an interview or a diary being suitable to identify any relation between ADHD and foods. Consequently, a diary or interview cannot be used to predict the child’s response to the diet or to detect foods that might trigger ADHD. Indeed, most children included in the ROMAN study (39/57 = 68%) had not previously followed some diet and participated because parents preferred not to start medication or because the effect of medication at home was disappointing. Nevertheless, in these children the FFD’s effect was equally high as in children already following an elimination diet.

No differences were found between practices, which may be attributable to the protocol that had to be meticulously followed: research has shown that adherence to protocols may improve clinical outcomes (19), while disregarding protocols may result in deficits in care (20, 21). The results in practice are comparable to the results of RCTs applying the FFD (9, 11, 14, 15).

In general practice, family structure is not assessed through questionnaires when applying the few-foods approach but is based on a parent-reported reflection on their parenting quality. Parenting may be qualified as “good” (i.e. most of the time consistent parenting and compliance with family rules) or “average” (some difficulties with consistent parenting and compliance with the family rules). It has been suggested that a stringent diet might affect family structure (22), thus underlying the behavioral improvements rather than the FFD. However, research has shown that the FFD did not affect family structure (23). Nevertheless, good family structure might be important to persevere in the FFD, especially in the reintroduction phase. Indeed, in the ROMAN study all families who reported an average family structure either did not start the reintroduction phase or stopped within 6 months, indicating that good family structure appears to be a prerequisite to adhere to the few-foods approach. It would be advisable to exclude families reporting an average family structure from starting the few-foods approach, or to initiate a supportive program to help these families through the reintroduction phase. Families with good family structure might also benefit from more comprehensive coaching, since for these families the long-lasting reintroduction phase is aggravating as well.

The few-foods diet is designed as a short-term diagnostic procedure to investigate whether or not food is a trigger of ADHD or ODD. It is a too restrictive diet to be used as a long-term intervention. The long-term effect of food has been investigated in previous studies applying the few-foods approach (14, 15): FFD responders proceeded with many introductions, one food a week. Subsequently, placebo foods were developed based on the incriminated foods according to the parental observations, and double-blind placebo-controlled trials were started to either confirm or refute the parental findings (14, 15). Although the exact duration is not mentioned in the papers, it seems obvious that reintroducing many foods, developing and testing placebo’s, and executing the double-blind trial would take at least 1.5 year, giving an indication of the long-term effect of food when applying the few-foods approach.

In the ROMAN study, 14 of 26 FFD responders who proceeded with the reintroduction phase still participated at 6 months, while 4 of 26 responders proceeded the introductions without expert supervision. All parents complained about the aggravating recurrence of behavioral problems during the reintroduction phase, inevitably leading to the re-elimination of the incriminated food, emphasizing the necessity for further research into the mechanisms of food in children with food-induced ADHD. Further research is especially important considering the substantial effect size, the applicability in practice, and the knowledge that research into the long-term effect of the most applied ADHD therapy to date, i.e., medication, showed disappointing results (7). Finding biomarkers, or pathways that may explain the effect of the FFD on ADHD, may eventually lead to new therapies and to redundancy of the few-foods approach.

Given the evidence for a microbiota-gut-brain interaction (24), further research into the underlying mechanisms of the FFD might specifically focus on the gut microbiome and the brain. Also, research has shown that inflammation might be a mechanism that is involved in ADHD (25). In light of the importance of scrutinizing the impact of the microbiome on ADHD (26), it is noteworthy that only recently a study was completed that specifically focussed on the impact of the FFD on the microbiota-gut-brain-axis and on the brain function in children with ADHD, i.e., the “Biomarker Research in ADHD: the Impact of Nutrition” (BRAIN) study (27). This study not only addressed the impact of the FFD on the gut microbiota, the metabolome, inflammation markers, and other parameters in urine, feces, and blood, but also investigated the impact of the FFD on neural activation in brain regions by means of functional MRI research.

In sum, the ROMAN results confirm the results of scientific research into the effect of the FFD and corroborate the existence of an ADHD subgroup with food-induced ADHD. Consequently, the few-foods approach has great potential for ADHD treatment: after defining whether a child belongs to the food-induced ADHD subgroup, the underlying triggers can be established and eliminated in FFD responders. Ultimately this approach may result in secondary prevention of ADHD. Considering the major impact of the FFD in children with ADHD it is timely to abandon the traditional viewpoint that ADHD is a single, symptom-based, diagnosis, and to include the FFD in both guidelines and diagnostic procedures. Stratifying between either “food-induced” or “classic” ADHD may result in personalized ADHD treatment, may improve ADHD health care and may lead to a decrease of medication use. In addition, acknowledgement and inclusion of the food-induced ADHD subgroup in future research into genetic background, phenotypes, endotypes, and/or prevention of ADHD may open a window of opportunity for children with ADHD.

## Conclusion

Our study shows that an FFD, applied in general practice by trained physicians, may have clinically relevant effects on ADHD and ODD, both in children with and without ADHD-medication or some elimination diet at start. Medication use decreased significantly in FFD responders. Applying the few-foods approach in practice may result in secondary prevention of ADHD. Further research into the mechanism(s) of food-induced ADHD is of vital importance to facilitate the few-foods approach or to replace this approach by an easier to apply intervention.

### Strengths and Limitations

The strengths of this study are the real-life setting, the data collection by different health care professionals (two physicians and one scientist practitioner) working in different practices, and the 6-months follow-up. A limitation is that no inventory was made of physical complaints, often co-occurring in children with ADHD, at start and end of the FFD. Another limitation is that this study is not a randomised controlled trial but an open study, which may lead to bias. However, post-RCT naturalistic studies in a heterogeneous population are important as well, in order to reflect on treatment management in clinical practice (28).

## Data Availability Statement

The datasets generated for this study are available at https://dans.knaw.nl/nl, doi: 10.17026/dans-xn4-6pjh.

## Ethics Statement

Ethical review and approval was not required for the study on human participants in accordance with the local legislation and institutional requirements. Written informed consent to participate in this study was provided by the participants’ legal guardian/next of kin.

## Author Contributions

Data was entered by each health care practice. LP and KF wrote the first draft of the manuscript. KF undertook the data analysis. LP, KF, JT and RRP contributed to the literature search, the interpretation of the data and the writing of the manuscript. All authors had full access to the data, approved the final version of the manuscript and decided to submit for publication.

## Conflict of Interest

LP has received honoraria for applying the FFD-approach in practice.

The remaining authors declare that the research was conducted in the absence of any commercial or financial relationships that could be construed as a potential conflict of interest.
